# Latent class analysis of association between occupational hazard factors and gamma-glutamyltransferase in automobile manufacturing workers: a cross-sectional study

**DOI:** 10.3389/fpubh.2026.1779616

**Published:** 2026-03-10

**Authors:** Huixia Ji, Dandan Liu, Ye Chen

**Affiliations:** Department of Occupational Disease, Nanjing Prevention and Treatment Center for Occupational Diseases, Nanjing, Jiangsu, China

**Keywords:** gamma-glutamyltranspeptidase, harmful gases, latent class analysis, noise, occupational hazard factors, organic toxins

## Abstract

**Background:**

Workers in the automobile manufacturing industry are potentially exposed to various occupational hazards that may impact serum gamma-glutamyltransferase (GGT) levels.

**Objective:**

This study aims to investigate the elevated serum GGT levels among workers in a automobile manufacturing plant and the impact of occupational hazards on GGT levels.

**Methods:**

Based on occupational health examination data collected from April to December 2024 from automobile manufacturing plant workers, this study classified nine major occupational hazards into five classes based on latent class analysis. Multivariate logistic regression was then applied to identify factors associated with elevated GGT levels, followed by subgroup analysis.

**Results:**

129 workers (17.9%) in this automobile manufacturing plant had elevated serum GGT levels. Primary occupational hazard exposures were classified into five distinct classes using latent class analysis: Class 1: low exposure, Class 2: noise exposure, Class 3: organic toxicants exposures, Class 4: dust exposures and Class 5: noise and harmful gases exposures. In multivariate logistic regression analysis, workers exposed to organic toxicants or noise were significantly more likely to have elevated GGT levels, with adjusted odds ratios (ORs) of 2.08 [95% confidence interval (CI): 1.09–4.16] and 2.65 (95% CI: 1.28–5.67), respectively. In subgroup analysis, workers exposed to Class 3 hazards in non-smoking and overweight-obese workers showed a significantly higher risk of elevated GGT levels, whereas workers exposed to Class 2 hazards showed a similar trend.

**Conclusions:**

The prevalence of elevated serum GGT levels among workers in automobile manufacturing plants is relatively high. Organic toxicants and noise may affect workers' serum GGT levels.

## 1 Introduction

Gamma-glutamyltranspeptidase (GGT) is a cell membrane enzyme that serves as a biomarker for liver injury and pathology. As part of glutathione (GSH) metabolism, GGT plays an important role in oxidative stress. Multiple diseases, such as metabolic syndrome, cardiovascular disease, type 2 diabetes, malignancies, respiratory disorders, and infectious diseases, are associated with elevated GGT levels (1). The increased expression of GGT is currently recognized as a biomarker for liver injury, cancer, and chronic inflammation (2). Monitoring GGT level fluctuations enables early identification of at-risk individuals and facilitates timely preventive interventions through occupational health risk assessment.

The level of GGT is commonly elevated in patients with steatotic liver disease, bile duct obstruction, and chronic alcohol consumption. Additionally, occupational exposures have been associated with increased GGT levels (3), suggesting liver injury may result from workplace hazards. Compared to ironers and cold-water washers, dry cleaners exposed to high concentrations of tetrachloroethylene have significantly higher GGT and alanine aminotransferase (ALT) levels (4). Perinatal exposure to certain pesticides, including glyphosate, promotes GGT activity in offspring's liver and blood through oxidative stress mechanisms, further supporting their hepatotoxic effects (5). GGT is a sensitive indicator of occupational liver disease. Monitoring GGT levels in occupational settings is therefore crucial for early diagnosis of liver injury. In China, GGT was formally incorporated as a diagnostic criteria for occupational chronic liver disease in 2025.

ALT and aspartate aminotransferase (AST) have been studied as liver function indicators in previous studies. During daily tasks in automobile manufacturing plants, workers may encounter various occupational hazards. This study is to evaluate the effects of occupational hazards on serum GGT levels among automobile manufacturing workers, thereby determining the underlying causes and guiding interventions.

## 2 Subjects and methods

### 2.1 Subjects

This study included workers from a large automobile plant who underwent occupational health examinations at the Nanjing Prevention and Treatment Center for Occupational Diseases between April and December 2024. The participants comprised two groups: (1) currently employed workers receiving their annual health surveillance, and (2) workers undergoing pre-departure health assessments. Specifically, the off-boarding assessments included in this study were those conducted within 3 months before leaving their jobs, ensuring that the measured biological indicators reflected recent occupational exposure. Inclusion criteria: (1) Workers officially recorded in the plant's occupational health monitoring system as being exposed to at least one occupational hazard, and (2) at least 1 year of cumulative work experience. Exclusion criteria: refusal to provide blood samples on the exam day. All female workers were excluded due to low numbers (*n* < 10). A total of 721 male workers were enrolled. The study was approved by the Ethics Committee of Nanjing Prevention and Treatment Center for Occupational Diseases (Approval No.: 2025-005) and was exempted from informed consent as it was retrospective.

### 2.2 Data collection

Demographic data, smoking history, alcohol consumption history, past medical conditions, body mass index [BMI = weight (kg)/height (m^2^)], and blood pressure readings were collected from all participants on the day of the physical assessment. Smoking history was defined as smoking at least three cigarettes per day for a continuous or cumulative period of 6 months or more. Alcohol consumption was defined as drinking at least once a week during the past year. Occupational exposure records were obtained directly from the plant's administrative department. The occupational exposure records provided included: the types of occupational hazards present in the workplace, the roster of exposed personnel, and job titles. The determination of exposure status was based on the plant's historical occupational hazard evaluation reports and task assignments, providing qualitative binary indicators (exposed/not exposed) for each hazard rather than quantitative exposure measurements. Laboratory evaluations included complete blood count, urinalysis, and liver and kidney function tests. Serum GGT levels were measured using the enzymatic rate method. Elevated GGT was defined as a level ≥50 U/L.

### 2.3 Statistical analysis

Statistical analysis was performed using R software (v4.4.3; R Core Team, Vienna, Austria). Categorical variables were presented as frequencies and percentages, and differences between groups were analyzed using the Chi-square (χ^2^) test. Skewed distributed data, are presented as median (interquartile range, Q1, Q3), and differences between groups were analyzed using the Mann-Whitney *U*-test. (1) Latent class analysis (LCA) of occupational risk factors: Potential risk factors with exposure rates below 10% were excluded, leaving nine occupational risk factors. Whether workers in the automobile plant were exposed to these factors was used as the indicator for LCA, which was conducted using the poLCA package in R. Model fit was evaluated using multiple criteria, including the Lo-Mendell-Rubin likelihood ratio test (LMRT) and the bootstrap likelihood ratio test (BLRT). A *p* value < 0.05 indicated that the k-class model provided a significantly better fit than the (k-1)-class model. Additional fit indices included the Akaike Information Criterion (AIC) and Bayesian Information Criterion (BIC), with lower values indicating better model fit. Entropy was used to assess classification precision, ranging from 0 to 1. Higher entropy values indicated greater classification accuracy, and an entropy value ≥0.80 suggested classification accuracy exceeding 90% (6–8). (2) Analysis of influencing factors: Elevated GGT levels during physical examination were used as the outcome variable (coded as: normal GGT = 0, elevated GGT = 1). Three logistic regression models were constructed: Model 1 included only the LCA-derived risk factor classes (with Class 1 as the reference), Model 2 added age and BMI as covariates, and Model 3 further incorporated smoking history (never smoked = 0, ever smoked = 1) and alcohol consumption history (never or occasional drinking = 0, regular drinking = 1). (3) Subgroup analysis: subgroup analyses were conducted stratifying the population by BMI (≥24 kg/m^2^ vs. < 24 kg/m^2^) and smoking history (ever vs. never) to evaluate potential effect modification. All statistical tests were two-sided, and a *p* value < 0.05 was considered statistically significant.

### 2.4 Validation of latent classes

Using available employment records (*n* = 578, 80.2% of the total sample), we examined the distribution of job titles within each class to establish the face validity of the latent class solution. Job titles were classified into 10 broad categories (e.g., Spray painting, Welding) based on task similarity and exposure profiles (see Supplementary Table S1 for detailed classification). Based on the frequency and proportion of each job category, we listed the top five specific job titles.

## 3 Results

### 3.1 Latent class analysis

**Figure 1:**
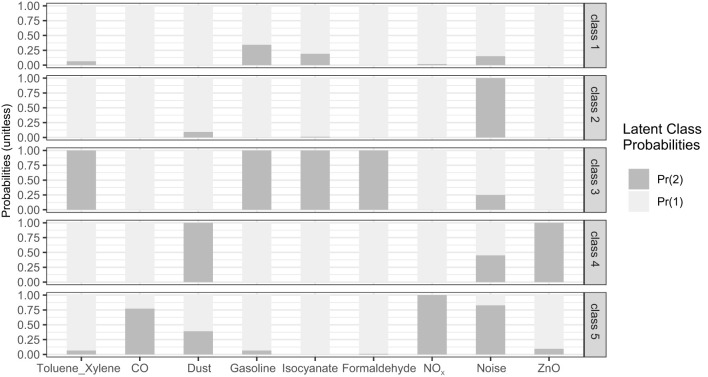
The conditional probability distributions of the five categories for each item.

Latent class analysis was performed incrementally by increasing the number of classes from one to six by using noise, dust, benzene series (toluene and xylene), gasoline, formaldehyde, zinc oxide, nitrogen oxides, carbon monoxide, and isocyanates as indicators. From one to six classes, both AIC and BIC values continuously decreased, LMR *p* values were all less than 0.001, and entropy values consistently exceeded 0.8, indicating high classification accuracy. The selection between the four-class and five-class models required careful consideration. The BLRT *p*-value for the five-class model was 0.188, which exceeds the 0.05 threshold. This result statistically favors the parsimony of the four-class model. However, the five-class solution was ultimately chosen for its practical interpretability and relevance to the study design. Specifically, the five-class model introduced a distinct Low Exposure Group (Class 1) that served as a clear baseline reference group. This group was not isolated in the four-class model, yet establishing such a reference group was essential for the subsequent analysis of occupational risk factors. Furthermore, the five-class solution maintained high classification quality, with an entropy of 0.957 and average posterior probabilities for each class exceeding 0.9 (0.982, 0.954, 1.000, 1.000, and 0.977). The six-class model was excluded because it yielded one class with a very low probability (0.97%), leading to instability in estimation. Details are provided in Table 1. Under the five-class solution, nine hazard factors were categorized and labeled based on their exposure patterns: Class 1: Low Exposure Group, Class 2: Noise Exposure Group, Class 3: Organic Toxicants Exposures Group, Class 4: Dust Exposure Group, and Class 5: Noise and Harmful Gases Exposure Group. Figure 1 shows the conditional probability distributions in the five classes.

**Table 1:** LCA of occupational exposure patterns to hazardous factors.

**Class**	**AIC**	**BIC**	**BLRT (*p*)**	**LMR (*p*)**	**Entropy**	**Category probability (%)**
1	6,401.759	6,442.984	—	—	—	100
2	4,916.436	5,003.468	< 0.001	< 0.001	1.000	13.31/86.69
3	4,316.711	4,449.550	< 0.001	< 0.001	1.000	23.58/63.11/13.31
4	3,920.520	4,099.165	0.052	< 0.001	1.000	22.19/15.26/49.24/13.31
5	3,812.934	4,037.385	0.188	< 0.001	0.957	13.31/22.19/18.03/31.21/15.26
6	3,758.826	4,029.084	0.186	< 0.001	0.964	31.21/19.00/0.97/13.31/13.31/22.19

AIC, Akaike information criterion; BIC, Bayesian information criterion; LMR, Lo-Mendell-Rubin; BLRT, bootstrap likelihood ratio test.

#### 3.1.1 Distribution of job titles within latent classes

In Table 2, the top five most frequent specific job titles are presented for each latent class. Statistically derived classes and actual job roles were highly concordant.

**Table 2:** Top five specific job titles within each latent class.

**LCA class**	**Class description**	**Representative job titles (*n*)**
Class 1	Low exposure group	Non-color painting robot maintenance (24) Process engineering (PE) maintenance (welding) (17) PE maintenance (final assembly) (15) Wax pump maintenance (9) General utilities (6)
Class 2	Noise exposure group	Press line unloading (17) Electrical power utilities (12) Power generation and refrigeration (11) PE maintenance (stamping) (10) Automated material handling (12)
Class 3	Organic toxicants	Touch-up and polishing (15) Color and clear coat robot maintenance (14) Paint surface adjustment (9) Defect spray painting (8) Wax pump maintenance (9)
Class 4	Dust exposure	Automated system maintenance workers (19) J72Y swing line production support (12) Side wall automated loading (12) Manual floor spot welding line (15) Grinding and polishing (10)
Class 5	Noise and harmful gases	Die maintenance (16) Engine compartment inspection (9) Bath solution management (8) Four-wheel alignment (8) Welding and grinding operations (8)

Class 1 (Low Exposure Group) was predominantly composed of maintenance and support roles, such as Non-color robot maintenance (*n* = 24) and PE (Process Engineering) maintenance (*n* = 17).

Class 2 (Noise Exposure Group) included workers from utility and stamping operations, such as Press line unloading (*n* = 17), Electrical utilities (*n* = 12), and Power generation/refrigeration (*n* = 11).

Class 3 (Organic Toxicants Exposure Group) was clearly characterized by direct painting and solvent-related tasks, including Touch-up/polishing (*n* = 15), Color/clear coat robot maintenance (*n* = 14), and Paint surface adjustment (9).

Class 4 (Dust Exposure Group) was largely represented by automation and production support roles, such as Automated maintenance workers (*n* = 19) and J72Y swing production support (*n* = 12), which are typically associated with environmental particulate matter exposure.

Class 5 (Noise and Harmful Gases Exposure Group) consisted of mixed-exposure roles, primarily Die maintenance (*n* = 16) and Engine compartment inspection (9), tasks that involve combined high-intensity noise, metal fumes, and physical hazards.

Job title information was missing for a portion of workers across all classes, which may limit the precision of this validation.

### 3.2 Demographic characteristics

One hundred and twenty nine workers (17.9%) in the automobile factory had elevated GGT levels, while 592 (82.1%) had normal GGT levels. The distribution of the five latent classes of occupational risk factors between the two groups was not statistically significant (*p* = 0.083). BMI differed statistically significantly between groups (*p* < 0.001). Age, smoking history, and alcohol consumption history were not significantly different (all *p* > 0.05). Detailed demographic characteristics are presented in Table 3. The demographic characteristics stratified by the five latent exposure classes are detailed in Table 4. No significant differences were observed in BMI, smoking history, or alcohol consumption among the classes (*p* > 0.05).

**Table 3:** Demographic characteristics of the normal group and GGT elevation group.

**Variables**	**All**	**Normal GGT**	**Elevated GGT**	***p*-Value**
	***N*** = **721**	***N*** = **592**	***N*** = **129**	
**LCA_class**				0.083
Class 1, low exposure	130 (18.0%)	116 (19.6%)	14 (10.9%)	
Class 2, noise	225 (31.2%)	182 (30.7%)	43 (33.3%)	
Class 3, organic toxicants	96 (13.3%)	72 (12.2%)	24 (18.6%)	
Class 4, dust	160 (22.2%)	133 (22.5%)	27 (20.9%)	
Class 5, noise + harmful gases	110 (15.3%)	89 (15.0%)	21 (16.3%)	
BMI	24.6 (22.6,26.7)	24.5 (22.2,26.4)	25.9 (23.7,28.1)	< 0.001
Age	37.0 (34.0,42.0)	37.0 (34.0,42.0)	36.0 (34.0,41.0)	0.133
**Alcohol**				0.074
No	667 (92.5%)	553 (93.4%)	114 (88.4%)	
Yes	54 (7.5%)	39 (6.6%)	15 (11.6%)	
**Smoking**				0.343
No	371 (51.5%)	310 (52.4%)	61 (47.3%)	
Yes	350 (48.5%)	282 (47.6%)	68 (52.7%)	

Data are presented as *n* (%) for categorical variables and Median (Q1, Q3) for continuous variables. BMI, body mass index; LCA, latent class analysis.

**Table 4:** Demographic characteristics of the study population stratified by the five latent exposure classes.

**Variables**	**Class 1**	**Class 2**	**Class 3**	**Class 4**	**Class 5**	***p*-Value**
	***N*** = **130**	***N*** = **225**	***N*** = **96**	***N*** = **160**	***N*** = **110**	
**Elevated GGT**						0.083
No	116 (89.2%)	182 (80.9%)	72 (75.0%)	133 (83.1%)	89 (80.9%)	
Yes	14 (10.8%)	43 (19.1%)	24 (25.0%)	27 (16.9%)	21 (19.1%)	
BMI	24.6 (22.6,27.0)	24.5 (22.2,26.4)	24.7 (23.2,27.4)	24.6 (22.2,26.6)	25.1 (23.2,26.8)	0.317
Age	37.0 (33.0,43.0)	36.0 (32.0,42.0)	37.0 (34.0,41.0)	36.0 (34.0,41.0)	39.0 (36.0,42.0)	0.058
**Alcohol**						0.771
No	120 (92.3%)	206 (91.6%)	87 (90.6%)	150 (93.8%)	104 (94.5%)	
Yes	10 (7.69%)	19 (8.44%)	9 (9.38%)	10 (6.25%)	6 (5.45%)	
**Smoking**						0.589
No	72 (55.4%)	121 (53.8%)	45 (46.9%)	77 (48.1%)	56 (50.9%)	
Yes	58 (44.6%)	104 (46.2%)	51 (53.1%)	83 (51.9%)	54 (49.1%)	

Data are presented as *n* (%) for categorical variables and Median (Q1, Q3) for continuous variables. BMI, body mass index.

### 3.3 Risk factors affecting GGT

An analysis of logistic regression was performed using GGT elevation status during medical examination as the dependent variable and occupational risk factors as the independent variable. The unadjusted model (Model 1) showed that Classes 2 and 3 had significantly increased risks of elevated GGT compared to Class 1 (reference group), with odds ratios (OR: 1.05–3.86) and 95% confidence intervals (Cls: 1.36–5.71). Model 2 adjusted for age and BMI, and the elevated GGT risk remained significantly higher in classes 2 and 3, with ORs (95% CIs) of 2.07 (1.09–4.13) and 2.69 (1.30–5.72). Model 3 further adjusted for smoking history and alcohol consumption history, yielding ORs (95% CIs) of 2.08 (1.09–4.16) for Class 2 and 2.65 (1.28–5.67) for Class 3. Table 5 shows detailed results.

**Table 5:** Logistic regression analysis of risk factors for GGT elevation in workers (odds ratios, 95% CI).

**Variables**	**Model 1**	**Model 2**	**Model 3**
**LCA_class**			
Class 1, low exposure	1.00 (reference)	1.00 (reference)	1.00 (reference)
Class 2, noise	1.96 (1.05–3.86, *p* = 0.042)^*^	2.07 (1.09–4.13, *p* = 0.031)^*^	2.08 (1.09–4.16, *p* = 0.031)^*^
Class 3, organic toxicants	2.76 (1.36–5.81, *p* = 0.006)^**^	2.69 (1.30–5.72, *p* = 0.008)^**^	2.65 (1.28–5.67, *p* = 0.010)^*^
Class 4, dust	1.68 (0.85–3.44, *p* = 0.141)	1.68 (0.84–3.47, *p* = 0.152)	1.70 (0.85–3.55, *p* = 0.141)
Class 5, noise + harmful gases	1.96 (0.95–4.14, *p* = 0.072)^†^	1.94 (0.93–4.15, *p* = 0.082)^†^	1.97 (0.94–4.24, *p* = 0.076)^†^
Age		0.98 (0.94–1.01, *p* = 0.166)	0.98 (0.94–1.01, *p* = 0.189)
BMI		1.18 (1.11–1.26, *p* < 0.001)^**^	1.19 (1.12–1.27, *p* < 0.001)^**^
Smoking			1.33 (0.89–1.98, *p* = 0.169)
Alcohol			1.76 (0.89–3.34, *p* = 0.090)^†^

Model 1: unadjusted, Model 2: Model 1 + age + BMI, Model 3: Model 2 + smoking + alcohol.

Significant codes: ^**^p < 0.01, ^*^p < 0.05, ^†^*p* < 0.1.

### 3.4 Subgroup analysis of factors associated with elevated GGT

**Figure 2:**
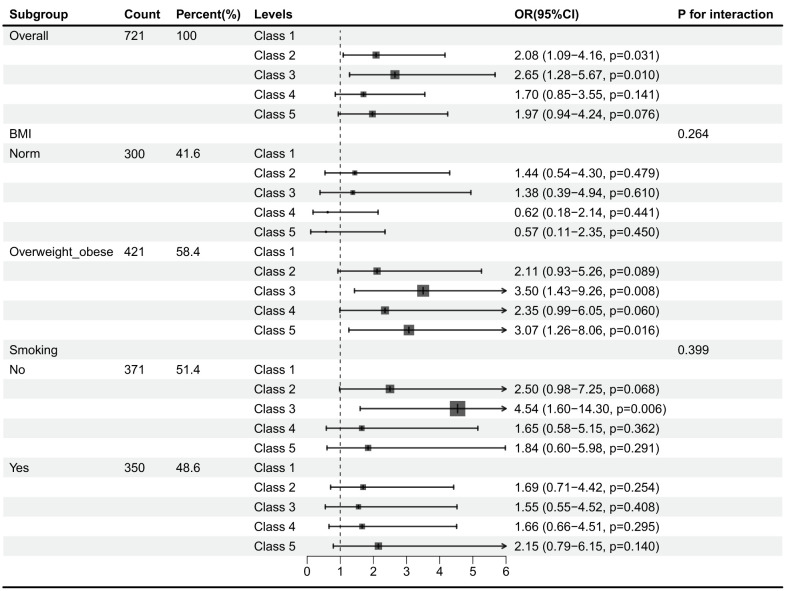
Subgroup analyses of factors influencing increased GGT in workers.

Overall, after adjusting for age, BMI, smoking history, and alcohol consumption history, Class 2 and Class 3 had significantly higher elevated GGT risks than Class 1. The subgroup analysis results are shown in Figure 2. Specifically, in overweight-obese and non-smoking groups, Class 3 showed a significantly increased risk of elevated GGT, while Class 2 showed a marginal trend.

## 4 Discussion

### 4.1 Association between exposure to organic toxins and elevated GGT levels in workers within the automotive manufacturing industry

Automotive manufacturing workers are frequently exposed to hazardous chemical substances, such as formaldehyde, gasoline, and isocyanates. In our study, 129 (17.9%) of 721 workers who participated in occupational health examinations showed elevated GGT levels. Based on LCA in conjunction with actual job titles, we identified the nine occupational hazards into five classes: Class 1: low exposure, Class 2: noise exposure, Class 3: organic toxicants exposures, Class 4: dust exposures and Class 5: noise and harmful gases exposures. After adjusting for potential confounding factors like age, BMI, smoking history, and alcohol consumption history, our study showed that individuals exposed to organic toxicants (Class 3) showed a significantly elevated risk of elevated GGT levels compared to those in Class 1. OR was 2.65 (1.28–5.67).

Gasoline is a volatile mixture containing benzene and aromatic hydrocarbons. Exposure to the BTEX group of compounds (benzene, toluene, ethylbenzene, and xylenes) is associated with elevated levels of GGT. Benzene, toluene, and ethylbenzene show relatively weaker correlations with increased GGT activity than xylene, which appears to be the primary contributor. GGT levels increase by 50.4% with every unit increase in xylene exposure (95% CI: 26.6%, 78.6%) (9). Exposure to chemical toxins is strongly correlated with elevated GGT levels. By promoting excessive reactive oxygen species (ROS) production, BTEX exposure leads to oxidative stress, lipid peroxidation, and hepatic injury. Moreover, BTEX exposure may trigger inflammatory responses that further damage the liver (9).

To date, there is no direct evidence that exposure to isocyanates and formaldehyde causes an increase in GGT levels. Formaldehyde exposure, however, can induce redox imbalance and oxidative damage (10). In addition, exposure to isocyanates decreases the levels of antioxidant enzymes, such as GSH and superoxide dismutase (11). Both chemical exposures can disrupt redox homeostasis, which is closely associated with the induction of oxidative stress. GGT serves as an early biomarker of oxidative stress (1), making its elevation a potential indicator. In this study, we found that mixed organic toxicant exposure was associated with elevated GGT; however, the specific contribution of individual agents (such as xylene) remains unclear. Further studies using cohorts with isolated exposure profiles are needed to clarify the independent effects of isocyanates, formaldehyde, and others.

### 4.2 The association between occupational noise exposure and combined exposure to noise and harmful gases with GGT levels in the automotive manufacturing industry

Noise, a prevalent occupational hazard, has been linked to elevated GGT levels. A significant increase in GGT risk was found after adjusting for potential confounders such as age, BMI, smoking history, and alcohol consumption history in the Class 2 noise exposure group compared to the Class 1 low exposure group. Noise and harmful gases combined in Class 5 did not result in elevated GGT levels.

Several studies have demonstrated that noise exposure increases oxidative stress biomarkers such as ROS and malondialdehyde (MDA), while simultaneously decreasing antioxidants such as GSH (12). Low-frequency noise exposure has also been observed (13). In response to oxidative stress, elevated GGT activity may represent a compensatory response. The hypothalamic-pituitary-adrenal axis activated by noise exposure may also exacerbate non-alcoholic steatotic liver disease (14), as well as influence lipid metabolism through the gut microbiota–gut–liver axis (15), potentially contributing to increased GGT. These findings suggest that GGT can serve as a biomarker for early liver injury and metabolic disturbances caused by noise.

When noise exposure is combined with harmful gases such as carbon monoxide and nitrogen oxides (Class 5), the trend of GGT elevation becomes non-significant (OR: 1.97, 95% CI: 0.94–4.24, *p* = 0.076). As of yet, no literature suggests that co-exposure to these two factors would counteract noise's effects on GGTs. A relatively small sample size may have limited statistical power, or it may reflect complex interactions among multiple occupational hazards. Further research with larger sample sizes is warranted. An OR of 3.07 (95% CI: 1.26–8.06, *p* = 0.016) is observed when overweight and obese individuals are exposed to this combined hazard environment. Metabolic syndrome is associated with elevated GGT levels (16). As overweight and obese individuals often exhibit metabolic disturbances and oxidative stress, they may be more susceptible to occupational hazards.

### 4.3 The role of oxidative stress in the automotive manufacturing industry

GSH is a thiol-containing antioxidant needed for many essential biochemical functions, including the regulation of vitamins D, E, and C, as well as detoxification of drugs. Further, GSH regulates mitochondrial metabolism and acts as a free radical scavenger to mitigate oxidative damage, thereby regulating oxidative stress (17). The metabolism of GSH is closely linked to GGT, an enzyme located on the extracellular membrane that catalyzes the hydrolysis of extracellular GSH, thereby releasing cysteine, which is a crucial precursor for the resynthesis of intracellular GSH. This mechanism maintains redox homeostasis and serves as an early biomarker of oxidative stress (1).

Multiple occupational health hazards are present in the automotive manufacturing industry, including organic toxins, noise, and dust. Occupational health interventions typically aim to reduce or eliminate exposure to these factors and improve personal protective measures. Exposure to organic toxins and noise may result in elevated GGT levels, possibly due to mechanisms related to oxidative stress, providing a novel perspective for occupational health interventions. Novel GGT inhibitors have been proposed as adjuncts to chemotherapy and liver disease treatment (2). Benfotiamine supplementation and aerobic exercise can also reduce oxidative stress markers, such as ROS and MDA, while increasing endogenous antioxidant defenses like GSH, thus alleviating noise-induced physiological damage. Further research is needed to determine whether exogenous GSH supplementation or novel GGT inhibitors can provide effective adjunctive therapies for occupational exposure-induced liver injury.

### 4.4 Limitations

The study has several limitations. Data from occupational health examinations do not include exposure duration and intensity. Additionally, GGT results were analyzed as a binary outcome, which may have reduced statistical power and information loss. As a result, dose-response relationships between exposure factors and GGT could not be verified, limiting the accuracy of the findings. Further, our occupational exposure portion analyzed only the correlation between mixed exposure and GGT levels, not the independent effects of each hazard factor. Moreover, only males were included due to the small number of female participants, making the conclusions less generalizable. Finally, smoking history and alcohol consumption were only treated as binary variables in this retrospective study. The coarse assessment method may have underestimated exposure. While alcohol consumption is known to be associated with elevated GGT levels (18, 19), we did not observe a significant association. This result may be attributed to the small number of drinkers in our sample (*n*=54). Among the 10 cases in Class 4 with alcohol consumption, none exhibited elevated GGT levels. Following the five-class LCA stratification, the absence of drinkers in certain classes made it impossible to calculate stable ORs.

## 5 Conclusion

Occupational exposure to organic toxins and noise in the automotive manufacturing industry is associated with elevated GGT levels. The combined exposure to noise and harmful gases does not show a significant effect on GGT, although a synergistic effect may exist among overweight and obese individuals. GGT may be useful as a biomarker for occupational health assessments, especially for those exposed to long-term organic toxins and noise. To safeguard workers' health, occupational health management strategies should incorporate comprehensive preventive and control measures. Large-scale prospective cohort studies could provide insight into occupational hazards and GGT, balance gender distribution and reduce confounding factors through accurate data collection. In the future, large-scale prospective cohort studies are warranted to precisely quantify the intensity and duration of single occupational exposures, ensure a balanced gender distribution, and refine the assessment of confounding factors, thereby further clarifying the association between occupational hazards and GGT.

## Data Availability

The raw data supporting the conclusions of this article will be made available by the authors, without undue reservation.
