# Supplementary material for: Latent class analysis of association between occupational hazard factors and gamma-glutamyltransferase in automobile manufacturing workers: a cross-sectional study
Source: Front Public Health. 2026 Mar 10;14:1779616. doi: 10.3389/fpubh.2026.1779616 (PMC13008871; doi:10.3389/fpubh.2026.1779616)
Supplement: Supplementary file 1 [file Table_1.docx]

Supplementary Table S1 Classification of specific job titles into broad job categories.

| Broad Job Category | Examples of Specific Job Titles |
| --- | --- |
| Spray painting/Coating | Spray painting, Touch-up, Paint mixing, Clear coat, Wax pump maintenance |
| Grinding/Cutting/Sheet metal | Grinding, Polishing, Cutting, Repair, Sheet metal adjustment |
| Welding/Stud welding | Spot welding, Arc welding, MIG, Laser welding, Stud welding |
| Maintenance/Technical | Robot maintenance, Equipment maintenance, Process Engineer, Mold maintenance |
| Assembly/General Assembly | Installation, Fastening, Interior assembly, Chassis assembly, Wire harness |
| Quality inspection/Testing | Inspection, Quality check, Audit, Measurement, Evaluation |
| Logistics/Transportation | Logistics, Forklift, Material handling, Transport |
| Management | Team leader, Supervisor, Manager, Master |
| Utilities/Plant Services | Electrical power, Refrigeration, Air compression, Energy supply |
| Others | Job titles not falling into the above categories |
